# Engineering of bioactive nanocomplexes on dental floss for targeted gingival therapy

**DOI:** 10.1002/btm2.10452

**Published:** 2022-12-20

**Authors:** Mayuka Nakajima, Nao Nakajima, Junling Guo, Samir Mitragotri

**Affiliations:** ^1^ John A. Paulson School of Engineering and Applied Sciences, Harvard University Cambridge Massachusetts USA; ^2^ Wyss Institute of biologically Inspired Engineering Boston Massachusetts USA; ^3^ Present address: Division of Periodontology, Department of Oral Biological Science Niigata University Graduate School of Medical and Dental Sciences Niigata Japan; ^4^ Present address: Division of Gastroenterology and Hepatology, Graduate School of Medical and Dental Sciences Niigata University Niigata Japan; ^5^ Present address: BMI Center for Biomass Materials and Nanointerfaces, College of Biomass Science and Engineering, College of Materials Science and Engineering Sichuan University Chengdu Sichuan China

**Keywords:** dental floss, periodontitis, polyphenol‐based bioactive nanocomplexes

## Abstract

Periodontitis induced by chronic subgingival infection is a ubiquitous disease that causes systemic inflammatory consequences and poses a negative impact on quality of life. The disease is treated and potentially prevented by patient's self‐care aimed at eliminating the oral pathogens from the region. Currently available products for interdental self‐care, including dental floss and interdental brush, have limited ability to prevent the disease. Here, we report a coated dental floss thread, termed “nanofloss,” which uses polyphenol‐based nanocoating to functionalize the floss thread with therapeutic agents. Multiple therapeutics can be integrated into the nanofloss including antibacterial small molecules and proteins. Flossing with nanofloss‐delivered therapeutic agents to the challenging subgingival region with long‐term retention even against the flushing action of the oral fluid in vivo. Our in vitro and in vivo studies demonstrate that chlorhexidine gluconate‐loaded nanofloss effectively treats the subgingival infection by *Porphyromonas gingivalis*. Collectively, the nanofloss offers a promising and easily usable tool for targeted self‐care of subgingival infection against periodontitis.

## 1 INTRODUCTION

Periodontitis, an irreversible inflammatory condition that causes a negative impact on oral health related quality of life, is a highly ubiquitous disease. Almost 50% of the adult population in the United States is affected by periodontitis, and the prevalence increases further with age.^1^, ^2^ Chronic inflammation causes destruction of the tooth‐supporting tissue and can lead to tooth loss thereby impacting speech, nutrition, and esthetic appearance.^3^ Periodontitis also has systemic inflammatory consequences, developing into other diseases such as cardiovascular disease, diabetes, and rheumatoid arthritis.^4^, ^5^ The major cause of periodontitis is the accumulation of bacterial plaque biofilm in the subgingival region, the gap between the tooth and the gingiva under the gingival margin, which causes dysbiosis of the oral microbiome and a destructive host inflammatory immune response.^6^ Therefore, removing the plaque is fundamentally important for the prevention and treatment of periodontitis. Moreover, since pathogens easily recolonize the gum pocket, daily plaque removal is required for effective treatment.

The gold standard of daily plaque removal is the combination of brushing and interdental cleaning.^7^ Normal toothbrushes have limited access to interdental regions and hence specially designed tools (e.g., interdental brush [IDB] and dental floss) are always required for adequate interdental cleaning. Among these, only IDB had yielded reduction of plaque and gingival inflammation, that too at a modest level.^8^ However, most interdental spaces, especially in young adults, are too small to be accessed by IDBs, which significantly hampers their widespread adoption. Although dental floss is commonly used in daily care and is universally accepted, systematic reviews and meta‐analyses have concluded that a majority of reported studies fail to demonstrate that flossing is generally effective in reducing plaque and moderating gum inflammation even when combined with toothbrushing.^8^, ^9^, ^10^ This originates from the fact that mechanical force is the primary mode of action of the dental floss. Therefore, it is highly desirable and clinically essential to further enhance the functionalities of dental floss beyond the simple mechanical mechanism through the integration of bioactive therapeutic molecules on the surface of floss thread.

**FIGURE 1:**
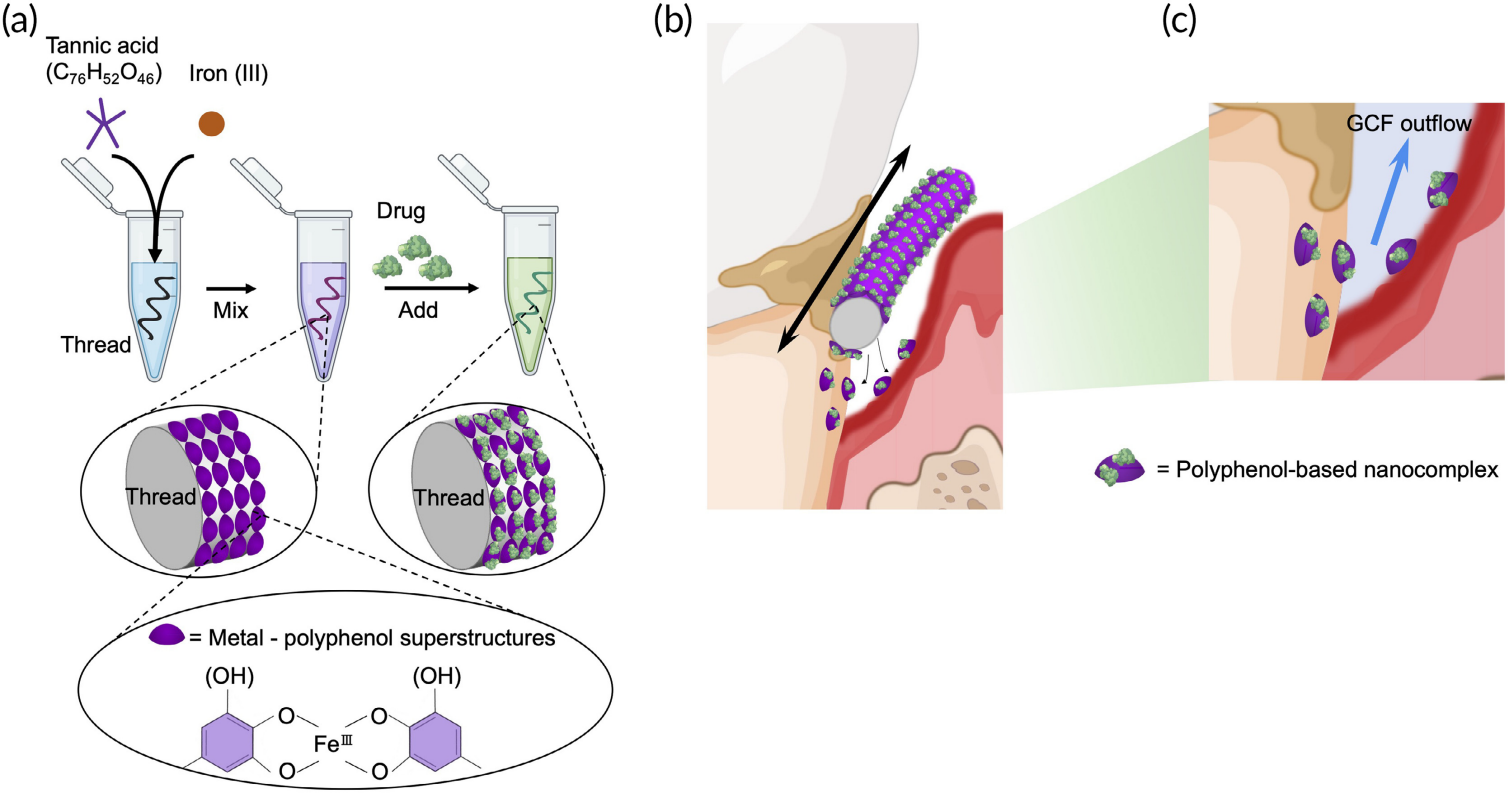
Schematic diagram of the “nanofloss” for an advanced care of the subgingival region. (a) Functionalization of silk thread as a drug carrier through rapid self‐assembly of metal‐polyphenol superstructures and drugs. Various type of agents can be loaded via interaction with polyphenol moieties. (b) Drug delivery with Nanofloss. Polyphenol‐based nanocomplexes are gradually transferred from nanofloss to the surface of teeth and gingiva under the subgingival region by flossing. (c) Sustained maintenance of drugs in the subgingival region. Adhesiveness of polyphenol helps retaining of drugs in the region against gingival crevicular fluid (GCF) outflow.

Here, we explored polyphenol‐based surface functionalization of the floss thread to load a series of bioactive molecules on its surface, referred to as “nanofloss,” to achieve targeted and sustained delivery of the active agents into the deep subgingival regions (Figure 1). Versatility of this approach was demonstrated by loading six different model agents onto the floss thread and their delivery into the target sites upon application of soft shear. Our results showed that the delivered polyphenol‐functionalized payload conjugates can be retained in the subgingival region for prolonged times even against flushing induced by oral fluids. In vitro studies showed that a commonly used dental disinfectant, chlorhexidine gluconate (CHG), can be integrated into nanofloss (CHG‐nanofloss) with high‐loading amount and long‐term sustained release in physiological conditions. In vivo results demonstrated that in a gum infectious rat model, CHG‐nanofloss demonstrated superior efficacy in eliminating the anaerobic pathogenic bacteria *Porphyromonas gingivalis* (*P. gingivalis*) from the subgingival region as compared with flossing with the conventional floss. These results demonstrated that polyphenol‐based functionalized floss provides a facile method to eliminate the formation of bacterial plaque. It also provides an effective daily tool for delivering therapeutic molecules such as antimicrobial‐ or anti‐inflammatory agents for improving the efficacy of flossing.

## 2 RESULTS

### 2.1 Fabrication of nanofloss and characterization

**FIGURE 2:**
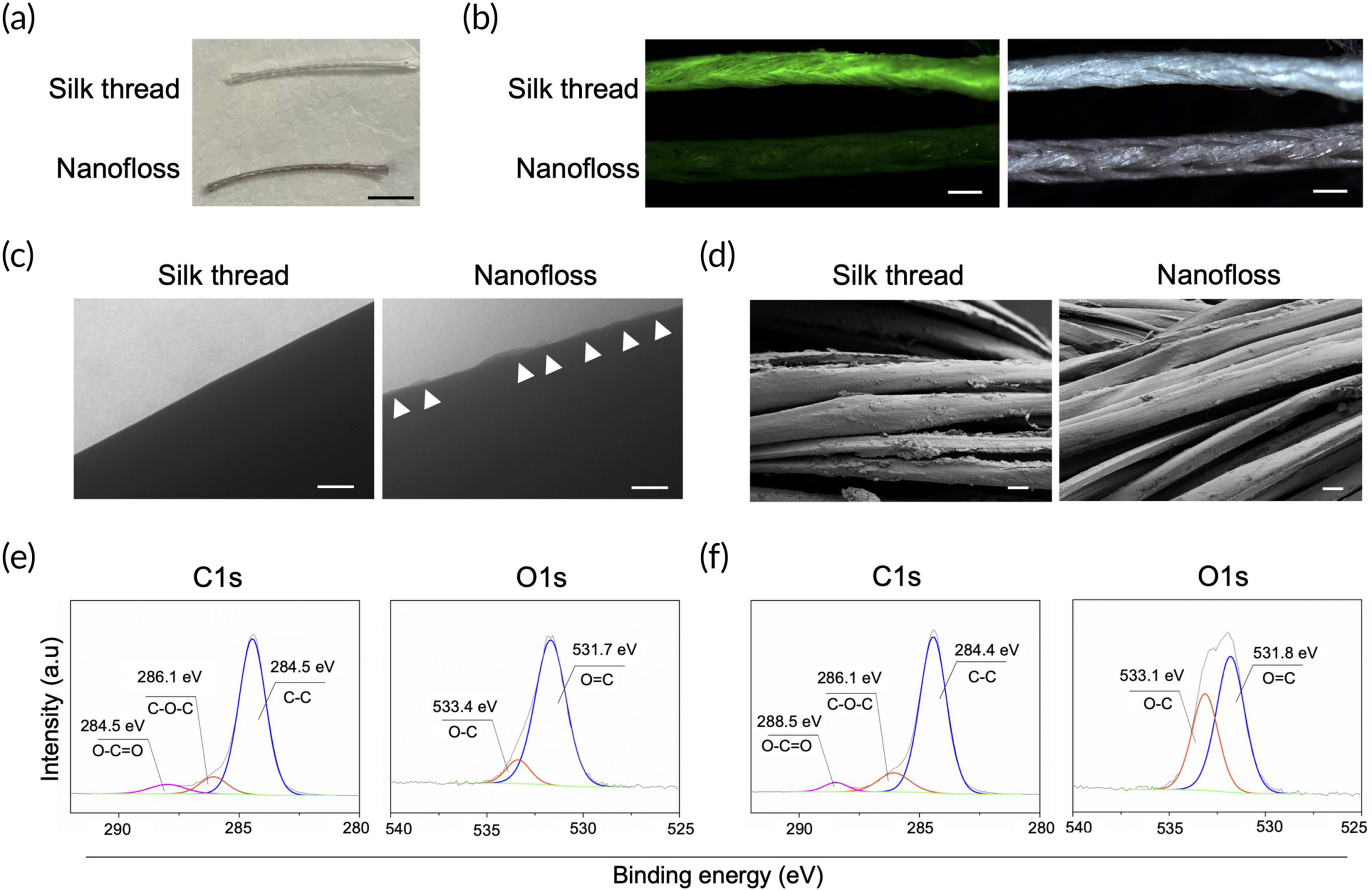
Physical characterization of nanofloss. A surface of commercially available thread (white) was engineered with metal‐polyphenol superstructures (purple). (a) Photograph of a naïve silk thread (white) and nanofloss (purple). Scale bar is 5 mm. (b) Fluorescent stereomicroscope images. Left: under GFP filter, right: bright field. Scale bars are 0.2 mm. (c) Transmission electron microscopy (TEM) images for a naïve silk thread and nanofloss. Scale bars are 100 nm. Arrowhead (►): metal‐polyphenol superstructures. (d) Scanning electron microscopy (SEM) images for a naïve silk thread and nanofloss. Scale bar are 10 μm (e). X‐ray photoelectron spectroscopy (XPS) analysis for a naïve silk thread. (f) XPS analysis for nanofloss

The surface of silk thread was coated using a simple dipping process. Commercially available threads were dipped in water and treated with tannic acid (TA), a natural polyphenol, and iron (III). The assembled metal‐polyphenol superstructures on the surface of the thread were visible to the eye as indicated by the purple color of the thread (Figure 2a). Observations under fluorescent stereomicroscope under green fluorescent protein (GFP) filter showed that intrinsic fluorescence of the original thread was quenched after the procedure, indicating that the threads were uniformly coated (Figure 2b). Uniform distribution of metal‐polyphenol superstructures on the thread was observed by transmission electron microscopy (TEM) (Figure 2c). The thread morphology was not altered by the assembled superstructures as confirmed by scanning electron microscopy (SEM) (Figure 2d). Furthermore, the x‐ray photoelectron spectroscopy (XPS) confirmed the presence of metal‐polyphenol superstructures on the threads (Figure 2e,f).

### 2.2 Integration of the drug payloads on nanofloss

**FIGURE 3:**
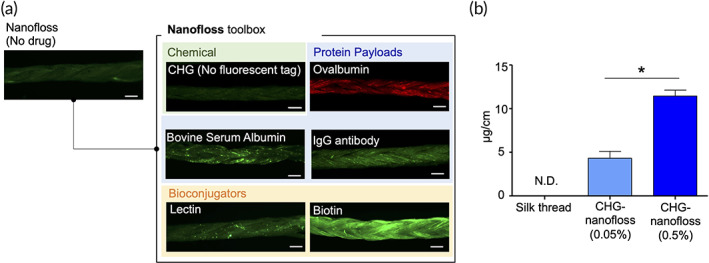
Versatility of nanofloss as a drug carrier. Drug payloads were loaded to the nanofloss through integration with the polyphenol moieties. (a) Fluorescence stereomicroscope images showing versatile toolbox of payloads for nanofloss. Six broad range of agents, which were conjugated with fluorescent tags (Alexa 488, Alexa 647, or FITC) were loaded on nanofloss via integration with the polyphenol moieties. Scale bars are 0.2 mm. (b) Concentration of CHG on nanofloss were measured by LC/MS (*n* = 4). Nanofloss were dipped into 0.05% or 0.5% of CHG solution to make CHG‐loaded nanofloss (CHG‐nanofloss). Significantly different (Mann–Whitney *U* test): *p < 0.05 Data are shown as mean ± SEM. CHG, chlorhexidine gluconate; LC/MS, liquid chromatography/mass spectrometer;N.D., not detected

Drug payloads were loaded on the nanofloss by adding them during the dipping process. Polyphenol‐based nanocomplexes were built rapidly by the integration of the payloads with the polyphenol moieties. Versatility of nanofloss as a drug carrier was demonstrated using six representative biomolecules, with different molecular sizes, charges, levels of hydrophobicity, and functionalities (Figure 3a). Chlorhexidine Gluconate (CHG), a disinfectant commonly used in mouthwash, was utilized as a representative small‐molecule chemical agent. CHG‐loaded nanofloss (CHG‐nanofloss) was prepared by soaking the floss into CHG solutions (0.05% or 0.5%). After removing the unbound agent by washing, concentrations of CHG on the CHG‐nanofloss were measured by liquid chromatography/mass spectrometry (LC/MS). LC/MS confirmed the presence of CHG on the nanofloss (Figure 3b). Concentration of CHG on the nanofloss increased with increasing concentrations of CHG in the loading solution, thereby confirming the ability to control CHG loading on the floss. CHG concentrations in CHG‐nanofloss contained higher concentrations of CHG than commercialized dental floss, which are impregnated with CHG (Table S1). The coating of the CHG payload on the thread is thin and is not expected to impact the mechanical strength of the thread.

### 2.3 Delivering small molecules by nanofloss

**FIGURE 4:**
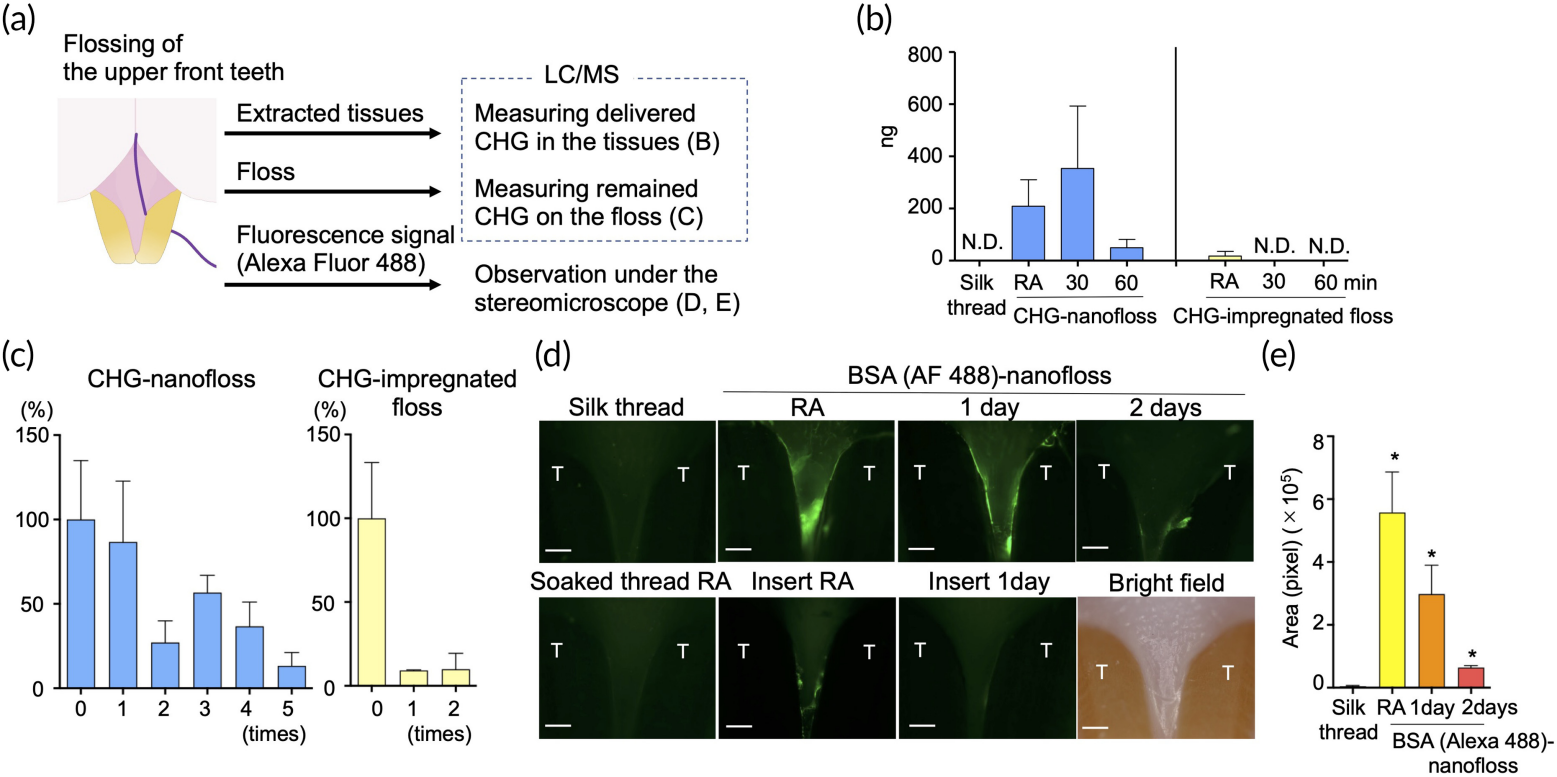
Delivering efficacy of nanofloss in vivo. Representative small (b, c) and large (d, e) molecules were loaded on to nanofloss to demonstrate the ability to deliver the molecules to subgingival region. (a) Schematic of in vivo delivering test. Deep subgingival regions of upper front teeth were flossed by drug‐loaded nanofloss, then three independent tests and analyses were performed. (b) The front teeth were flossed by CHG‐nanofloss or commercialized CHG‐impregnated floss, and the concentrations of delivered CHG in the tissues were measured by LC/MS (*n* = 4). Statistical analysis (Mann–Whitney *U* test) was performed between CHG‐nanofloss and CHG‐impregnated floss. There was no statistical difference right after the flossing. (c) Frequency test: Concentrations of remained CHG on CHG‐nanofloss or the commercialized CHG‐impregnated floss after flossing were measured by LC/MS (*n* = 3). The concentrations before flossing were set as 100%. (d) Representative fluorescence stereomicroscope images for interdental papilla of upper front teeth after flossing with BSA (Alexa Fluor 488)‐nanofloss. As a negative control, naïve silk thread was soaked into BSA solution, rinsed in water, then applied for the front teeth (Soaked thread RA). As a positive control, BSA solution was insert into the gingival sulcus using a needle (Insert RA and 1 day). Scale bars are 0.3 mm. (e) Green area on the fluorescence stereomicroscope images were measured by ImageJ. Significantly different (Mann–Whitney *U* test): versus noncoated, *p < 0.05. CHG, chlorhexidine gluconate; LC/MS, liquid chromatography/mass spectrometer; N.D., not detected; T, tooth; RA, right after the application

The ability of nanofloss to deliver agents into the subgingival region was demonstrated using both small and large molecules (Figure 4a). CHG was utilized as a representative small‐molecule drug. CHG‐nanofloss was inserted into the deep subgingival region of rat upper front teeth and the teeth were then flossed. The concentrations of delivered CHG in the tissues were measured by the LC/MS. Substantial amounts of CHG were delivered by flossing with CHG‐nanofloss, and the delivered CHG was detectable over 60 min (Figure 4b). On the other hand, in the case of commercial CHG‐impregnated dental floss, CHG was detected only in the tissue right after flossing. Amounts of CHG delivered from the floss into the gums decreased gradually over five applications, while most of the CHG on the commercial CHG‐impregnated dental floss was delivered in the first flossing (Figure 4c).

### 2.4 Delivering large molecules by nanofloss

Bovine serum albumin (BSA) conjugated with Alexa Fluor (AF) 488 dye was used as a model macromolecule drug. The fluorescent signal in the subgingival region of rat the upper front teeth was observed at various time points by fluorescent stereomicroscope (Figure 4d,e). Clear green line that matched with gingival sulcus, the narrow gap between the tooth and gingiva, confirmed that BSA was successfully delivered throughout the subgingival region by BSA‐nanofloss. Needle‐insertion‐mediated delivery of BSA into the sulcus faded away within 1 day. However, strong signal was observed in the sulcus after 1 day after flossing with BSA‐nanofloss. No fluorescent signal was observed after flossing with BSA soaked naïve silk thread.

### 2.5 Long retention of agents in subgingival region

Gingival crevicular fluid (GCF), an outflow under the gingival margin, is a major factor that makes it difficult for the administered agents to remain in the subgingival region.^11^ However, as shown in Figure 4, both small and large molecules were maintained for prolonged times in the region against the flow. To reveal the underlying mechanisms of prolonged retention, rubbing test of nanofloss was carried out first. Nanofloss, which is visibly purple, turned white after the rubbing test, while the rubbed site turned purple, thus demonstrating that the polyphenol‐based nanocomplexes were transferred from the nanofloss to the application site (Figure S1A,B). Next, the retention of the polyphenol‐based nanocomplexes against the flow was observed by a slide‐based test (Figure S2). A microscope glass slide was rubbed with BSA (AF 488)‐nanofloss or BSA soaked naïve silk thread, followed by washing with running water (500 μl, 100μl/s). The flow rate of water was much higher than previously reported values of GCF (0.008 μl/s).^12^ The fluorescent signal was well preserved after exposure to running water, demonstrating that polyphenol‐mediated nanocomplexes adhered to the slide against the water flow. Taken together, these data support the transfer of polyphenol‐based nanocomplexes from the nanofloss to the subgingival region.

### 2.6 Therapeutic efficacy of CHG‐nanofloss

**FIGURE 5:**
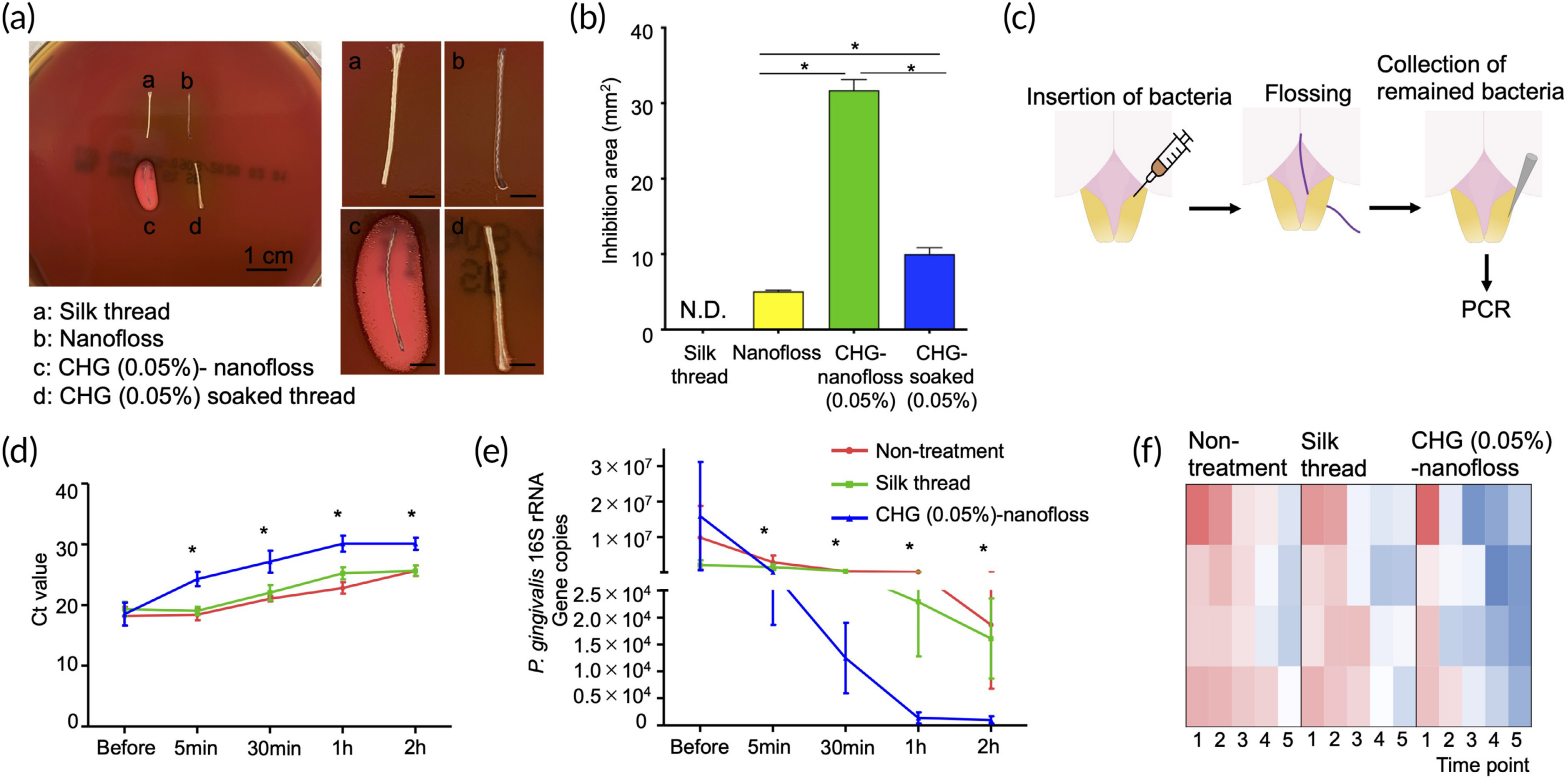
Antimicrobial effects of CHG‐nanofloss in vitro and in vivo. In vitro antimicrobial test. (a) Disk diffusion test. Threads were placed on the bacteria cultured plate. Bactria growth‐inhibition were observed after incubation under the anaerobic condition for several days. Images on the right side are enlarged. Scale bars are 0.2 mm. (a) Naïve silk thread, (b) nanofloss, (c) nanofloss‐loaded CHG (0.05%) (CHG [0.05%]‐nanofloss), (d) silk thread soaked in CHG (0.05%) solution (CHG [0.05%] soaked thread). (b) Bacteria growth‐inhibition area around the threads were measured by ImageJ (*n* = 6). Significantly different (Kruskal–Wallis test followed by Bonferroni correction): *p < 0.017. Data are shown as mean ± SEM. In vivo antimicrobial test using subgingival infectious model. (c) Schematic of making the infectious model, treatment, and analysis. Infected subgingival region of rat upper front teeth were treated by naïve silk thread or CHG (0.05%)‐nanofloss. The fluid samples were collected from the sites before and after the flossing. (d) Ct values and (e) gene copies of the *P. gingivalis* 16S rRNA in the fluid samples were analyzed using RT‐PCR (*n* = 4). Significantly different (Kruskal–Wallis test): *p < 0.05. Data are shown as mean ± SEM. (f) Heatmap for gene copies of the *P. gingivalis* 16 S rRNA in the fluid samples. Red: high, blue: low number of the gene. Time point: 1: Before flossing, 2: 5 min, 3: 30 min. 4: 1 h, 5: 2 h after the flossing. CHG, chlorhexidine gluconate; N.D., not detected

Since bacterial infection is the most important cause of periodontitis, antimicrobial activity of CHG‐nanofloss was evaluated in vitro and in vivo. In vitro efficacy was studied using disk diffusion test. Growth inhibition of *Porphyromonas gingivalis*, the major periodontopathic bacteria, was measured on the agar plate (Figure 5a,b). Nanofloss itself, without the drug, exhibited slight antimicrobial efficacy; however, the effect was dramatically enhanced by loading CHG on to nanofloss. Unlike aerobic bacteria, periodontopathic bacteria exist in the deep subgingival region, which is anaerobic. To mimic these conditions, the bacterial suspension was inserted into the bottom of the gingival sulcus then the sites were treated by CHG‐nanofloss in vivo (Figure 5c). The bacterial DNA was isolated from the sulcus and quantified by quantitative PCR (qPCR). Flossing with naïve silk thread did not yield any improvement compared with the untreated site. However, the number of bacteria decreased significantly after flossing with CHG‐nanofloss (Figure 5d–f).

## 3 DISCUSSION

Periodontitis requires lifelong maintenance by both patients and dental professionals.^13^ Even after receiving active periodontal therapy, residual deep periodontal pockets, the deep subgingival region, represent a significant risk factor for the relapse of inflammation leading to further disease progression.^14^, ^15^ Despite substantial research, the current treatment options fail to control the progression of the disease. In this study, we report a modular strategy of engineering silk thread surface taking advantages of self‐assembled polyphenol‐based nanocomposites, referred to as nanofloss, for an advanced self‐care of subgingival region. This technology enables the delivery and retention of various agents in the subgingival region, and CHG‐loaded nanofloss effectively treated the bacteria infection.

Polyphenol‐based bioactive nanocomplexes leverage the interactions between polyphenol‐metal and polyphenol‐agents. This polyphenol‐based strategy for surface‐functionalization is simple and inexpensive, and thus it has been utilized to coat various substances, including particles and cells, for various applications including drug delivery, wound healing, and imaging^16^, ^17^, ^18^, ^19^, ^20^, ^21^ and hence was selected for design of nanofloss. Successful coating of the thread surface opens another interesting application of this technology.

Another valuable feature of the polyphenol‐based nanocomplexes described here is that they could be transferred from the thread to the tissue surface. The presence of polyphenol in the nanocomplexes of active agents enabled their retention on the tissue surface.

Dental floss is an ideal tool for targeted drug delivery to the subgingival region for prevention and treatment of periodontitis. Dental floss is already a common tool for personal care and is easily accessible to the broad population. Topical delivery of drugs for the treatment of periodontitis is a major challenge due to limited tissue penetration. Current products intended for periodontitis need to be inserted into periodontal pockets directly by dentists, which requires patients to visit the clinic on a regular basis.^11^, ^22^, ^23^ Although dental floss is commonly used for tooth cleaning, little has been published on its use as a topical drug carrier. Kaewiad et al. showed that dental floss impregnated with antibacterial agent inhibit bacterial growth on agar plates,^24^ and Muniz et al. demonstrated the effect of chlorhexidine impregnated dental floss on supragingival plaque reduction in clinical trials.^25^, ^26^ However, neither study demonstrated actual drug delivery into the subgingival region by the floss. Further, the antimicrobial effect under the gingival margin was unclear. Boese et al. designed coated dental floss and demonstrated delivery efficacy of the floss to porcine gum pockets ex vivo^27^; however, it was still unclear whether the delivered drug was able to tolerate the outflow of GCF. The present study uniquely demonstrates that nanofloss delivers therapeutic agents into the subgingival region and they remain stable in the delivered area in living animals. This study also clearly demonstrates that both small and large molecules can be delivered to the desired site by flossing with nanofloss. Retaining agents, especially small molecules, in the subgingival region poses a significant challenge,^28^ which can be addressed by nanofloss. Adhesiveness of polyphenols^29^, ^30^ assists in retention of transferred polyphenol‐based nanocomplexes onto the gingival tissues and tooth surfaces. Based on its ability to coat and deliver multiple agents, nanofloss offers a versatile tool to treat periodontitis.^31^ Chlorhexidine is a commonly used active ingredient in mouthwash. Hence, it was used as a representative drug in this study. In future, additional drugs could be incorporated into nanofloss to diversify the payloads. Current commercial chlorhexidine‐based mouthwashes contain as much as 0.2% chlorhexidine, whereas the nanofloss carries about 10 μg of chlorhexidine per cm of the thread. A 10 cm nanofloss thus contains about 100‐times less chlorhexidine than a 10 ml dose of mouthwash. While this provides a foundation for safety of the nanofloss‐approach, additional detail studies are necessary to assess long‐term safety of nanofloss. Since the use of chlorhexidine against *P. gingivalis* has been extensively studied in the literature, mechanisms of its efficacy against *P. gingivalis* were not studied here. Such studies should also be performed in future endeavors.

## 4 CONCLUSION

This study reports a natural polyphenol‐based strategy to functionalize floss with a range of bioactive molecules (referred to as nanofloss) for targeted delivery to the deep pocket sites of the subgingival region. Polyphenol‐functionalized molecules can be retained in the pathological sites against oral flush. Nanofloss enabled long‐term retention of dental disinfectant CHG in vivo and achieved a highly effective treatment to eliminate the growth of anaerobic pathogenic bacteria *P. gingivalis* in subgingival region. From a therapeutic point of view, this functionalized floss could offer an active treatment of subgingival infection through targeted delivery of antibacterial and anti‐inflammatory agents, which addresses one of the most prominent challenges in periodontitis therapy.

## 5 MATERIALS AND METHODS

### 5.1 General materials

TA, iron(III) chloride hexahydrate (FeCl_3_·6H_2_O), biotin‐4‐fluorescein, and FITC (Fluorescein)‐conjugated lectin were purchased from Sigma‐Aldrich (MO, USA). AlexaFluor 488 BSA, AlexaFluor 647 ovalbumin (OVA), custom‐designed oligonucleotide, and Fast SYBR Green Master Mix were procured from Thermo Fisher Scientific Inc (MA, USA). Blood agar plate, glutaraldehyde was ordered from VWR (PA, USA). The following materials were obtained from the manufacturers as shown below: 4‐0 silk thread: MANI (Tochigi, Japan), Tris solution pH 8: Santa Cruz Biotechnology (TX, USA), Formvar/Carbon Film on 400 mesh on grids: EMS (PA, USA), CHG: Spectrum Chemical Mfg. Corp. (NJ, USA), Alexa Fluor 488 Goat anti‐rat IgG Antibody: BioLegend (CA, USA), *P. gingivalis* W83: ATCC (VA, USA), modified Gifu Anaerobic Medium (GAM) broth: HyServe (Uffing, Germany), QIAamp DNA Blood Mini Kit: QIAGEN (MD, USA).

### 5.2 Fabrication of nanofloss

All solutions were freshly prepared for immediate use. Silk thread was soaked in 500 μl of water. The 20 μl of TA in water (24 mM) was added and the mixture was vortexed briefly. Immediately, 20 μl of FeCl_3_∙6H_2_O in water (22 mM) was added and vortexed for 10 s. After 5‐min incubation, the pH was then raised by adding 500 μl of Tris buffer (100 mM, pH 8), followed by further vortex. The thread was rinsed with water to remove excess TA and FeCl_3_∙6H_2_O.

### 5.3 TEM imaging

Small pieces of a naïve silk thread and nanofloss were sandwiched between two TEM grids. TEM images were obtained on a JEM‐1400 TEM (JEOL, MA, USA) with an operation voltage of 80 kV.

### 5.4 SEM imaging

A naïve silk thread and nanofloss were placed on SEM holders and coated with platinum/palladium (80/20) using an EMS150T turbo‐pumped sputter coater/carbon coater (EMS, PA, USA). SEM images were obtained on a ZEISS FESEM Ultra‐55 field‐emission scanning electron microscope (SEISS, Potsdam, Germany), operating at an accelerating voltage of 2 kV.

### 5.5 X‐ray photoelectron spectroscopy

XPS measurement was completed on the Nexsa XPS system from Thermo Scientific. The probe for the measurement was aluminum K‐α X‐ray line with energy at 1.4866 keV and x‐ray spot size was set at 150 μm. XPS data were taken after the chamber pressure was sitting at 5 E‐8 mBar or lower. Flood gun, which supplies both low‐energy electron and ion, was used throughout the entire experiment for sample surface charge compensation. Both survey spectrum (wide) and high‐resolution scan (narrow) were collected on both a naïve silk thread and nanofloss. The atomic percentages of each element and the carbon and oxygen peak deconvolution were performed by using the Thermo Scientific Avantage software. For survey spectrum, which was used to generate atomic percentages of each element, the scan was completed by taking average of five scans with passing energy at 200 eV and dwell time at 10 ms. For high‐resolution scans, the data were collected by taking average of 10 scans with passing energy at 50 eV and dwell time at 50 ms for both carbon 1s photoelectron line, which was further deconvoluted into C—C (at 284.5 eV, B.E.), C—O—C (at 286.10 eV, B.E.) peaks and O—C=O (at 288.0 eV, B.E.), and oxygen 1 s photoelectron line, which was further deconvoluted into O=C (at 531.7 eV, B.E.) and O—C (at 533.1 eV, B.E.) peaks. The XPS instrumental error for atomic composition is ±1%, and the accuracy of the C1s and O1s peak fitting are ±2%.

### 5.6 Loading drugs to nanofloss

A wide range of agents were used to demonstrate the versatility of nanofloss as a drug carrier. Drug solutions (i.e., proteins, Biotin, Lection) (20–40 μg/ml) and CHG solutions (0.05% and 0.5%) were prepared. Nanofloss were soaked into 500 μl of the solutions for 15 min and rinsed with water to remove excess agents. The drug loaded to nanofloss was observed by fluorescent stereomicroscope (Axio Zoom.V16; Zeiss, Potsdam, Germany).

### 5.7 Liquid chromatography/mass spectrometry

The concentration of CHG on nanofloss and delivered to the subgingival tissues were measured by LC/MS as described previously with slight modifications.^32^ The measurements were undertaken by Agilent 1290 Infinity II LC/MSD XT, an ultra‐high‐performance liquid chromatography analysis system equipped with an electrospray ionization‐mass spectrometry detector, using a ZORBAX RRHD C18 column (2.1 × 100 mm, 1.8 μm, Agilent) with a UHPLC guard column, ZORBAX RRHD C18 column (2.1 × 5 mm, 1.8 μm, Agilent). The mobile phase was composed of methanol—ammonium formate (10 mM)—formic acid (56:44:0.2, v/v/v) (pH 3.5) and was delivered isocratically at a flow rate of 0.5 ml/min. The column temperature was controlled at 40°C and the injection volume was 5 μl. The peak at 1.2 min was identified as the peak. Calibration curves were prepared, consisting of seven samples covering a concentration range of 4.4–250 μg/mL of CHG. Sample preparation for the LC/MS analysis was carried out as described below. CHG‐loaded nanofloss or commercialized floss with CHG were placed into 50% methanol/water mixture and shaken 15 min, while animal tissues (teeth and gingiva) were shaken in the 50% methanol overnight. Afterward, the mixture was filtered by 0.2 μm nylon syringe filter for the analysis.

### 5.8 Animals

Male Wistar rats weighing 276–300 g were acclimated in an animal room for 3 days and provided with regular chow and sterile water throughout the experiment. All experiments were performed according to the approved protocols by the Institutional Animal Care and Use Committee of the Faculty of Arts and Sciences, Harvard University, Cambridge.

### 5.9 Drug delivery to subgingival region

The delivering ability of nanofloss and pharmacokinetics of the delivered drug in the tissues were studied in live rats using two different types of agents, CHG and BSA. CHG (0.05%)‐nanofloss or commercialized floss immersed with CHG were inserted into the deep subgingival region of rats' upper front teeth. After 5‐times flossing, the front teeth and gingival tissues were collected at several time points and placed into vials, which contain 50% methanol for LC/MS study to measure delivered CHG in the tissues. The LC/MS study was performed as described above.

Upper front teeth were flossed by BSA (Alexa 488)‐nanofloss and the interdental area was observed by fluorescent stereomicroscope chronologically up to 2 days. Naïve silk thread was used as a negative control. In addition, naïve silk thread was soaked into BSA solution, rinsed in water, and then applied for the front teeth. As a positive control, BSA solution was insert into the gingival sulcus using a needle. The stereomicroscope images were split by color channels by ImageJ (NIH). Bright green fluorescent areas on the stereomicroscope images, which were split by color channels were measured by ImageJ (NIH).

### 5.10 Frequency test

Frequency of drug release from nanofloss was studied. Rats' upper front teeth were flossed by 10 cm of CHG‐nanofloss or commercialized floss with CHG for one to five times. After the flossing, each thread was placed into 50% methanol and the concentrations of CHG that remained on the threads were measured by the LC/MS. The LC/MS preparation is as described above. Concentrations of CHG before flossing were set as 100%.

### 5.11 Bacteria culture

For all bacterial studies, 25 μl of *P. gingivalis* W83 from frozen stock was added to 5 ml modified GAM broth and incubated in an anaerobic jar for 48 h at 37°C until reached to 1 × 10^9^ CFU (colony forming units)/ml. AnaeroPack‐Anaero was placed inside the jar to maintain anaerobic conditions during the culture period.

### 5.12 In vitro antimicrobial test

Disk diffusion test was conducted to study in vitro antimicrobial effect of CHG‐nanofloss. Naïve silk thread, nanofloss, CHG (0.05%)‐nanofloss, and silk thread soaked in CHG (0.05%) solution (CHG [0.05%] soaked thread) were obtained for this experiment. All threads were rinsed in water and dried out before using. *P. gingivalis* suspension (1 × 10^8^ CFU/700 μl) was spread onto blood agar plate. The threads were placed onto the agar gel and incubated for 8 days under the anaerobic condition. Bactria growth‐inhibition zone around the threads were measured by ImageJ.

### 5.13 In vivo antimicrobial test

Following the insertion of the bacteria suspension (1 × 10^9^ CFU/ml) into the gingival sulcus of front teeth by syringe, the front teeth were flossed by the threads once. The bacterial DNA in the sites were taken by sterile paper points size #45 chronologically before and after the flossing. The paper points were placed in 200 μl of PBS, and the bacterial DNA was isolated using the QIAamp DNA Blood Mini Kit. Total 5 μl of DNA suspension was used for qPCR. qPCR was performed on a CFX96 RT PCR (Bio‐Rad, CA, USA) using a Fast SYBR Green Master Mix. The custom‐designed oligonucleotide sequences for the *P. gingivalis* 16 S rRNA gene were as listed below. Forward: AGGCAGCTTGCCATACTGCG, and reverse: ACTGTTAGTAACTACCGATGT. The Ct values obtained from the qPCR were converted to gene copy numbers to estimate the amount of bacterial genomes. All experiments were performed using the protocols approved by the institutional committee at Harvard University.

### 5.14 Statistical analysis

All data are presented as mean ± SEM. Statistical analyses were performed using GraphPad Prism (GraphPad Software, Inc., San Diego, CA, USA). Comparison between two groups was conducted using Mann–Whitney *U* test. Comparisons among multiple groups were conducted using Kruskal–Wallis test followed by Bonferroni post hoc test. Statistical significance was assumed at *p < 0.05, **p < 0.01.

## AUTHOR CONTRIBUTIONS

**Mayuka Nakajima:** Conceptualization (equal); data curation (lead); formal analysis (lead); funding acquisition (equal); investigation (lead); methodology (lead); writing – original draft (lead); writing – review and editing (equal). **Nao Nakajima:** Methodology (equal). **Junling Guo:** Conceptualization (equal); methodology (equal); writing – review and editing (supporting). **Samir Mitragotri:** Conceptualization (equal); project administration (lead); resources (lead); supervision (lead); writing – review and editing (equal).

## CONFLICT OF INTEREST

The authors declare that they have no competing interests here.

## Supporting information


**Appendix S1:** Supporting Information

## Data Availability

The data that supports the findings of this study are available in the article and the supplementary material of this article.
