# Supplementary material for: Engineering of bioactive nanocomplexes on dental floss for targeted gingival therapy
Source: Bioeng Transl Med. 2022 Dec 20;8(2):e10452. doi: 10.1002/btm2.10452 (PMC10013826; doi:10.1002/btm2.10452)
Supplement: Supplementary file 1 — Appendix S1: Supporting Information [file BTM2-8-e10452-s001.docx]

**Supplementary Materials for**

**Engineering of bioactive nanocomplexes on dental floss for targeted gingival therapy**

Mayuka Nakajima, Nao Nakajima, Junling Guo*, and Samir Mitragotri*

This file includes:

Table. S1. Concentrations of CHG on nanofloss

Figure S1. Transferability of polyphenol-based nanocomplexes (purple) from thread to the delivered site.

Figure S2. Polyphenol-based nanocomplexes retained delivered site against water flow.

**Table. S1. Concentrations of CHG on nanofloss.** Concentration of CHG on nanofloss were measured by LC/MS. Nanofloss were dipped into 0.05 or 0.5% of CHG solution to make CHG loaded nanofloss (CHG-nanofloss). The concentrations in each CHG -nanofloss were compared with that in the commercialized dental floss which is impregnated with CHG. Significantly different (Mann-Whitney U test): vs commercialized *P < 0.05 Data are shown as mean ± SD (n=4). CHG: Chlorhexidine gluconate, LC/MS: Liquid Chromatography/Mass Spectrometer

**Figure S1. Transferability of polyphenol-based nanocomplexes (purple) from thread to the delivered site.** Nanofloss were rubbed against white paper to observe color changes in nanofloss and the paper. (A) Stereomicroscope image of nanofloss before/after the rubbing test. Color in nanofloss (purple) was changed to white after rubbing test, indicating polyphenol-based nanocomplexes (purple) was removed from the surface of nanofloss. Scale bar is 0.5 mm. (B) Photo for the paper after rubbing with nanofloss or naïve silk thread. Purple color on the white paper after rubbing with nanofloss showing presence of polyphenol-based nanocomplexes on the paper. Scale bar is 5 mm.

**Figure S2. Polyphenol-based nanocomplexes retained delivered site against water flow.**

Bovine serum albumin (BSA) conjugated with Alexa Fluor 488 dye was loaded to Nanofloss or naïve silk thread. The edge of microscope glass slide was flossed with the threads, then the flossed sites were washed by water. Stereomicroscope images were taken under bright field or GFP filter before and after washing. BSA was delivered by BSA-nanofloss on the slide, and the delivered BSA was retained against water flow. Scale bars are 1 mm.
